# Comparative Pharmacological Profiling of Psychotherapeutic Drugs Reveals a Functional Taxonomy Based on Direct Inhibition of Smooth Muscle Excitability

**DOI:** 10.3390/ph19040645

**Published:** 2026-04-21

**Authors:** María Jesús Castrillejo, Alfonso Velasco, Juan F. Mielgo-Ayuso, Jesús Pérez, Manuel Garrosa, Carlos Alberto Rodríguez-Arias, Diego Fernández-Lázaro

**Affiliations:** 1 Department of Cell Biology, Genetics, Histology and Pharmacology, Faculty of Medicine, University of Valladolid, 47005 Valladolid, Spain; mariajesus.castrillejo@estudiantes.uva.es; 2 Department of Cell Biology, Genetics, Histology and Pharmacology, Faculty of Medicine, Institute of Neurosciences of Castile and Leon (INCYL), University of Valladolid, 47005 Valladolid, Spain; fonch38@hotmail.com; 3 Degeneration and Regeneration Laboratory, Institute of Neurosciences of Castile and Leon (INCYL), University of Valladolid, 47005 Valladolid, Spain; manuel.garrosa@uva.es (M.G.); carodrigueza@saludcastillayleon.es (C.A.R.-A.); 4 Faculty of Health Sciences, University of Burgos (UBU), 09001 Burgos, Spain; 5 Advanced Research in Integrative Physiology for Life Research Group (IAFIV), University of Burgos (UBU), 09001 Burgos, Spain; 6 Department of Medicine, Faculty of Medicine, Institute of Biomedical Research (IBSAL), University of Salamanca, 37007 Salamanca, Spain; jesusperez@usal.es; 7 Area of Histology, Faculty of Medicine, University of Valladolid, 47005 Valladolid, Spain; 8 Neurobiology Research Group, University of Valladolid, 47005 Valladolid, Spain; 9 Neurosurgery Department, Hospital Clínico Universitario de Valladolid, Institute of Neurosciences of Castile and Leon (INCYL), 47003 Valladolid, Spain; 10 Area of Histology, Faculty of Health Sciences, University of Valladolid, 42004 Soria, Spain; 11 Biomedical Research Institute of León (IBIOLEÓN), University Hospital Complex of León, 24071 León, Spain

**Keywords:** psychotherapeutic drugs, smooth muscle excitability, K^+^-induced depolarization, functional pharmacological profiling, autonomic side-effect liability, ex vivo assay

## Abstract

**Background:** Autonomic side effects are a major determinant of tolerability for many psychotherapeutic drugs. While often attributed to receptor-mediated mechanisms, the potential contribution of direct modulation of smooth muscle excitability remains poorly characterized at a comparative pharmacological level. **Methods:** A systematic comparative pharmacological profiling of a broad panel of psychotherapeutic drugs (antidepressants, antipsychotics, and anxiolytics) was conducted using a standardized ex vivo model. Potassium chloride (KCl, 105 mM) was used to induce depolarization-dependent contraction in three isolated smooth muscle preparations (rat uterus, rat vas deferens, and guinea-pig ileum). Inhibitory potency (IC_50_), dose-dependency, and tissue consistency were integrated to define functional inhibitory profiles. **Results:** Psychotherapeutic drugs exhibited marked heterogeneity in their ability to inhibit K^+^-induced smooth muscle contraction. Integrative analysis stratified compounds into four distinct functional profiles: (i) High Inhibitory Liability (e.g., nortriptyline, paroxetine), characterized by low micromolar IC_50_ values and dose-dependent inhibition across multiple tissues; (ii) Non-Selective Inhibition (e.g., flunarizine, cinnarizine), showing consistent but dose-independent inhibition; (iii) Tissue-Dependent Inhibition (e.g., risperidone, reboxetine); and (iv) Minimal Inhibition (e.g., moclobemide). Agents classified within the High Inhibitory Liability profile correspond to drugs known to carry a higher clinical burden of autonomic adverse effects. **Conclusions:** This study reveals a previously underrecognized pharmacodynamic dimension of psychotherapeutic drugs and establishes a comparative functional taxonomy based on their direct, non-receptor-mediated inhibition of smooth muscle excitability. The identified profiles provide a mechanism-informed framework for contextualizing autonomic side-effect liability and may support improved safety evaluation in psychotherapeutic drug development.

## 1. Introduction

The clinical utility of psychotherapeutic drugs is frequently limited by autonomic nervous system-mediated adverse effects, including orthostatic hypotension, constipation, urinary retention, and xerostomia [1,2]. These effects contribute substantially to poor treatment adherence, increased morbidity, and reduced quality of life [3,4,5]. Although such adverse effects are reported across multiple psychotherapeutic drug classes, including antidepressants, antipsychotics, and anxiolytics [6,7,8], traditional explanations have focused primarily on antagonism of peripheral muscarinic, adrenergic, or histaminergic receptors [1,2,7]. This receptor-centric framework, however, provides an incomplete pharmacodynamic account of autonomic liability [7,8,9], and the perceived burden of these effects independently reduces patient quality of life [5].

Beyond classical receptor blockade, many psychotherapeutic agents exhibit membrane-stabilizing or local anesthetic-like properties, reflecting a direct interaction with excitable cell membranes [10,11,12]. These properties are commonly attributed to interference with voltage-gated sodium and calcium channels, which represent shared molecular determinants of depolarization-dependent excitability across excitable tissues [13,14,15]. Although such channel-level interactions are well documented in cardiac and neuronal tissues [10,11,16], their implications for smooth muscle function—a key effector system of autonomic responses—have received comparatively limited attention.

In vascular, gastrointestinal, and genitourinary smooth muscle, excitation–contraction coupling critically depends on membrane depolarization and calcium influx through voltage-dependent Ca^2+^ channels [17,18]. Accordingly, direct modulation of depolarization-dependent excitability constitutes a fundamental determinant of smooth muscle tone and contractile behavior [17,18]. Despite this clear physiological basis, a systematic comparative characterization of the capacity of psychotherapeutic drugs to directly inhibit smooth muscle contraction—and its potential contribution to autonomic side-effect liability—is currently lacking. Importantly, the present study was designed to specifically address direct pharmacodynamic effects of psychotherapeutic drugs on smooth muscle excitability, independently of systemic pharmacokinetic factors. To this end, an ex vivo experimental approach was selected, allowing the assessment of intrinsic drug–tissue interactions while excluding confounding influences related to absorption, distribution, metabolism, and elimination.

High extracellular potassium (K^+^)-induced smooth muscle contraction provides a standardized, non-receptor-mediated experimental approach to interrogate this direct pharmacodynamic mechanism [19,20]. By bypassing neurotransmitter receptors, K^+^-induced depolarization isolates a drug’s intrinsic ability to interfere with the final common pathway of excitation–contraction coupling, primarily via voltage-dependent Ca^2+^ influx [19,20]. However, a systematic comparative pharmacological profiling of a broad panel of psychotherapeutic drugs using this approach has not been performed, leaving the field without a functional taxonomy based on this direct mechanism.

We therefore hypothesized that psychotherapeutic drugs exhibit a heterogeneous spectrum of activity in inhibiting K^+^ -induced smooth muscle contraction that can be stratified into distinct and reproducible functional profiles. The objectives of this study were: (i) to perform a systematic comparative pharmacological profiling of a broad panel of psychotherapeutic drugs using potassium-induced depolarization in three ex vivo smooth muscle preparations; and (ii) to integrate inhibitory potency, dose-dependency, and tissue-consistency data to establish a functional classification that may provide a mechanistic framework for contextualizing their reported autonomic side-effect liability.

## 2. Results

### 2.1. Inhibitory Potency of Psychotherapeutic Drugs on Potassium-Induced Smooth Muscle Contraction

The inhibitory effects of the psychotherapeutic drugs on KCl (105 mM)-induced smooth muscle contraction are summarized in Table 1. Among the compounds that produced concentration-dependent inhibition, the half-maximal inhibitory concentrations (IC_50_) were predominantly within the micromolar range. However, substantial heterogeneity was observed in inhibitory potency, concentration–response behavior, and tissue consistency.

Across compounds, inhibitory effects were absent at lower concentrations and emerged above compound-specific threshold concentrations. Above these thresholds, drugs exhibited either concentration-dependent inhibition, allowing IC_50_ determination, or dose-independent inhibition, characterized by a lack of proportional increase in effect with increasing concentration.

Several drugs, such as nortriptyline, clotiapine, and paroxetine, exhibited low micromolar or submicromolar IC_50_ values in one or more preparations, indicating a potent inhibitory interaction with depolarization-dependent contraction. In contrast, other compounds, including moclobemide, displayed no detectable effect within the concentration range tested in the preparations where they were evaluated.

A distinct and notable pattern was observed for a subset of drugs, including the known calcium channel modulators flunarizine, cinnarizine, and dotarizine. For these agents, inhibition occurred in a consistent but dose-independent manner across all tested preparations, precluding the calculation of classical IC_50_ values and suggesting a non-canonical, likely non-competitive, mechanism of action.

The experimental approach employed a tiered strategy; all drugs were tested in at least one smooth muscle preparation, with select compounds profiled in additional tissues to assess consistency (see Table 1 for complete testing matrix). The experimental approach employed a tiered strategy; all drugs were tested in at least one smooth muscle preparation, with select compounds profiled in additional tissues to assess consistency (see Table 1 for complete testing matrix). This design enabled the identification of distinct inhibitory profiles despite pragmatic constraints related to experimental feasibility. This strategy also reflects ethical considerations aimed at minimizing animal use, as well as practical constraints related to preparation stability during ex vivo recordings.

### 2.2. Functional Pharmacological Profiling Based on Inhibitory Patterns

Based on an integrated analysis of inhibitory potency, concentration–response behavior, and consistency across the tested smooth muscle preparations, the psychotherapeutic drugs were stratified into four distinct functional pharmacological profiles (Table 2).

The High Inhibitory Liability profile (Profile 1) comprised compounds that produced clear, concentration-dependent inhibition with low micromolar or submicromolar IC_50_ values in two or more smooth muscle preparations. Drugs within this group, including nortriptyline, clotiapine, paroxetine, nefazodone, and melitracen, reproducibly suppressed K^+^ -induced contraction across tissues, indicating a robust interference with depolarization-dependent excitability.

A second profile, designated Non-Selective Inhibition (Profile 2), was defined by a consistent pattern of inhibition that emerged only above a minimal effective (threshold) concentration but did not exhibit a classical sigmoidal concentration–response relationship across multiple preparations. This behavior, which precludes classical sigmoidal curve fitting and reliable IC_50_ estimation in the affected tissues, was observed for flunarizine, dotarizine, and cinnarizine. Although cinnarizine exhibited a calculable IC_50_ in rat uterus, its predominant dose-independent inhibition in rat vas deferens and guinea-pig ileum aligned it functionally with this profile. This difference reflects tissue-specific concentration–response behavior, as cinnarizine produced a graded, concentration-dependent inhibition in rat uterus, whereas in rat vas deferens and guinea-pig ileum inhibition occurred without a progressive concentration–response relationship, precluding reliable IC_50_ estimation.

The Tissue-Dependent Inhibition profile (Profile 3) encompassed drugs whose inhibitory effects were restricted to specific preparations. Risperidone inhibited contraction in rat vas deferens and guinea-pig ileum (the latter in a dose-independent manner) but showed no detectable effect in rat uterus, whereas reboxetine produced concentration-dependent inhibition exclusively in rat uterus, with no effect in the other preparations tested.

Finally, a Minimal or Absent Inhibition profile (Profile 4) was defined for compounds that failed to modify K^+^-induced contraction in the preparations evaluated. Moclobemide consistently showed no inhibitory activity in rat uterus or vas deferens and was therefore classified within this category.

Together, this functional profiling reveals a reproducible stratification of psychotherapeutic drugs based solely on their direct effects on potassium-induced smooth muscle depolarization, independent of receptor-mediated mechanisms.

### 2.3. Tissue Consistency of Inhibitory Responses

The consistency of inhibitory effects across different smooth muscle preparations is summarized for a representative subset of compounds in Table 3. Certain agents, notably nortriptyline and the calcium channel modulators cinnarizine and flunarizine, inhibited K^+^-induced contraction in all preparations in which they were evaluated, indicating broad and tissue-independent inhibitory activity.

In contrast, other drugs exhibited clear tissue selectivity. Risperidone inhibited contraction in rat vas deferens and guinea-pig ileum but showed no detectable effect in rat uterus, whereas reboxetine produced inhibition exclusively in rat uterus. Moclobemide consistently failed to modify K^+^-induced contraction in the preparations tested.

Collectively, these findings demonstrate that psychotherapeutic drugs display distinct and reproducible patterns of tissue consistency in this ex vivo model, reinforcing the functional stratification and providing a structural basis for subsequent mechanistic interpretation.

Importantly, the observed heterogeneity in inhibitory potency, dose-dependency, and tissue consistency enabled a comparative pharmacological profiling of psychotherapeutic drugs based solely on their functional interaction with potassium-induced depolarization. Inter-individual variability was reflected in the confidence intervals of IC_50_ estimates, whereas the qualitative inhibitory profiles remained consistent across independent tissue preparations.

### 2.4. Graphical Summary of Inhibitory Profiles

**Figure 1:**
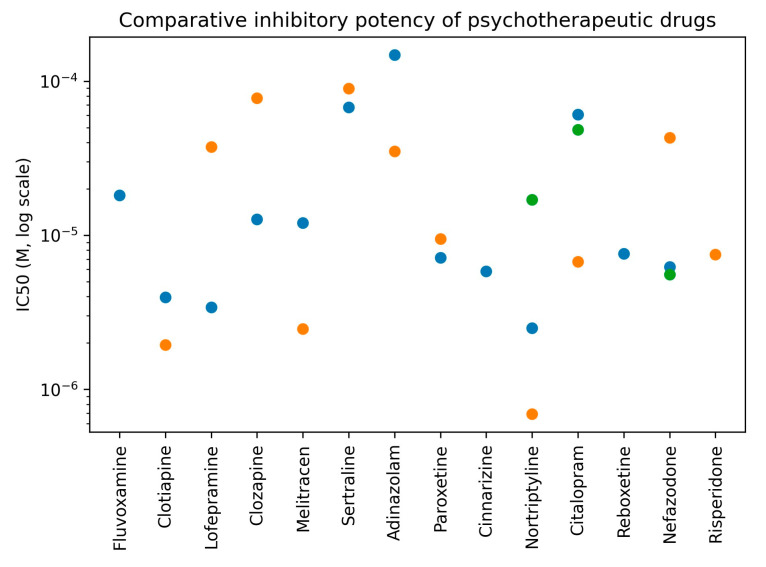
Comparative inhibitory potency (IC_50_) of psychotherapeutic drugs on K^+^-induced smooth muscle contraction in rat uterus, rat vas deferens, and guinea-pig ileum. IC_50_ values are displayed on a logarithmic scale. Data points are color-coded according to smooth muscle preparation. Only compounds exhibiting concentration-dependent inhibition are shown.

To facilitate visual comparison of inhibitory potency across compounds and tissues, IC_50_ values derived from concentration–response experiments are summarized graphically in Figure 1 to enable comparative visualization of inhibitory potency across compounds and tissues.

This graphical representation highlights the marked heterogeneity in inhibitory potency among psychotherapeutic drugs and supports the quantitative differences summarized in Table 1.

**Figure 2:**
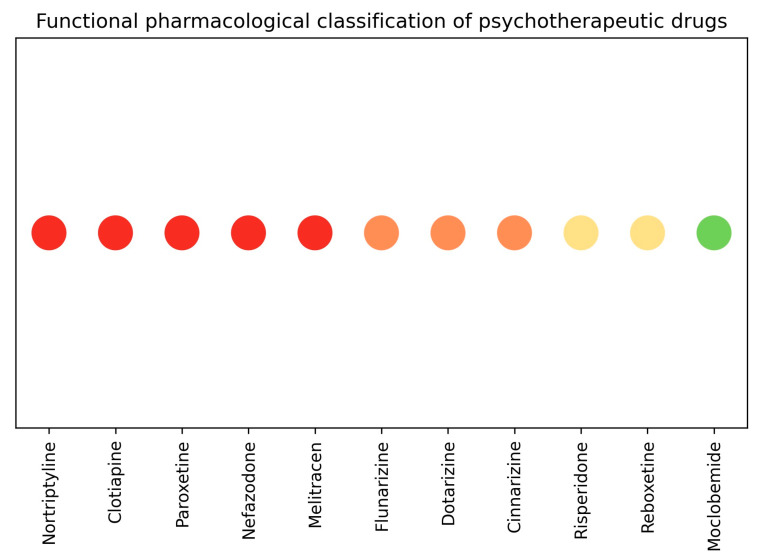
Functional pharmacological classification of psychotherapeutic drugs based on inhibitory potency, dose-dependency, and tissue consistency. Colors indicate functional profiles: High Inhibitory Liability, Non-Selective Inhibition, Tissue-Dependent Inhibition, and Minimal or Absent Inhibition.

Based on an integrated analysis of inhibitory potency, dose-dependency, and tissue consistency, psychotherapeutic drugs were stratified into four functional pharmacological profiles. This classification is summarized graphically in Figure 2.

This visual representation provides a concise overview of the functional profiles identified and facilitates comparison of direct smooth muscle inhibitory behavior across drug classes.

**Figure 3:**
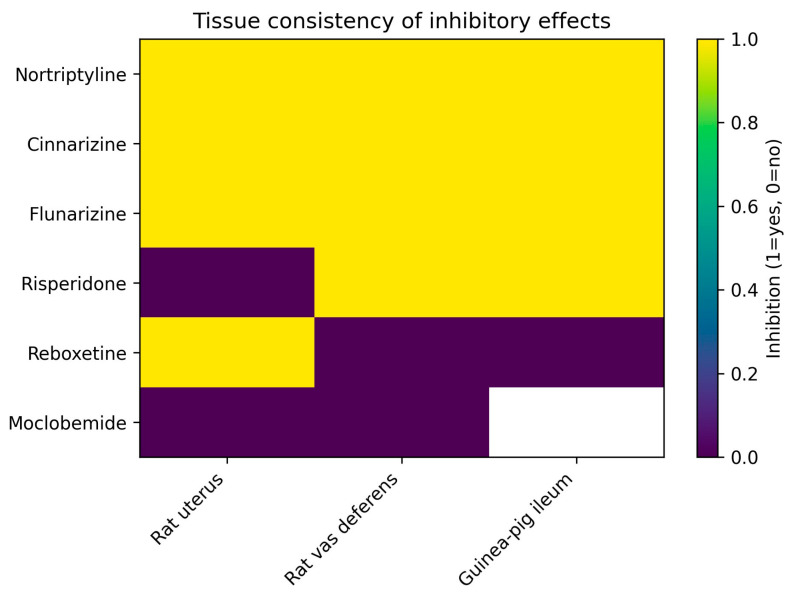
Tissue consistency of inhibitory effects of selected psychotherapeutic drugs on K^+^-induced smooth muscle contraction. Colors indicate presence or absence of inhibition across smooth muscle preparations; white color indicates not tested (NT).

To further illustrate the consistency of inhibitory effects across smooth muscle preparations, tissue-specific responses for representative compounds are summarized in Figure 3.

The patterns illustrated in Figure 3 reinforce the tissue-dependent behavior observed for specific compounds and support the use of multiple smooth muscle preparations for comparative functional pharmacological profiling.

**Figure 4:**
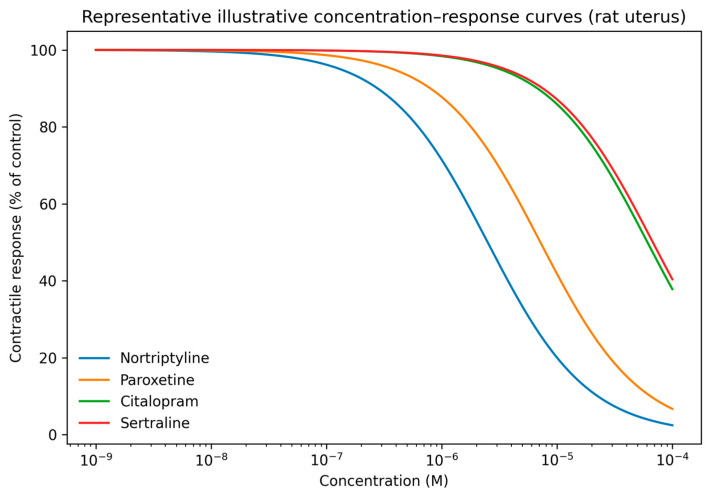
Representative illustrative concentration–response curves for selected psychotherapeutic drugs in rat uterus. Curves were generated using a standard sigmoidal model parameterized with experimentally determined IC_50_ values and identical slope assumptions. These curves are provided for qualitative visualization only and do not represent original experimental recordings.

To further facilitate interpretation of concentration–response behavior, representative illustrative curves reconstructed from experimentally determined IC_50_ values are shown in Figure 4 for selected compounds in rat uterus. These curves provide a visual approximation of relative inhibitory potency and profile shape across compounds.

Complete sets of illustrative curves across all tissues are provided in Supplementary Figures S1–S3.

## 3. Discussion

To our knowledge, this study provides the first systematic comparative pharmacological profiling of a broad panel of psychotherapeutic drugs based on their direct, non-receptor-mediated inhibition of smooth muscle excitability. This integrated analysis revealed substantial heterogeneity in their ability to inhibit K^+^-dependent depolarization–contraction coupling, enabling a novel stratification into four distinct functional profiles based on inhibitory potency, dose-dependency, and tissue consistency. While original experimental concentration–response curves are not included in the main text, illustrative curves derived from IC_50_ values are provided in Supplementary Figures S1–S3, and the graphical summaries presented herein provide a transparent visualization of the quantitative and qualitative patterns that underpin the proposed functional classification. Because the primary aim of the present study was comparative functional classification rather than detailed pharmacometric characterization of individual compounds, graphical summaries derived from experimentally determined IC_50_ values were considered sufficient to support the proposed taxonomy and its mechanistic interpretation.

The functional stratification derived from our integrated analysis (Table 2) not only categorizes the observed inhibition but also reveals divergent pharmacodynamic behaviors. The High Inhibitory Liability profile (Profile 1), characterized by low micromolar IC_50_ values and dose-dependent inhibition across multiple preparations, is pharmacologically consistent with a potent interaction with voltage-gated sodium (Na^+^) and calcium (Ca^2+^) channels [11,12,13,16,21], which have demonstrated that several antidepressants and antipsychotics directly inhibit voltage-gated Na^+^ currents and, in some cases, Ca^2+^ entry in excitable tissues.

Such behavior aligns with the well-documented membrane-stabilizing properties of several tricyclic antidepressants and certain antipsychotics [21,22,23]. These studies describe the membrane-stabilizing and local anesthetic-like properties of several psychotherapeutic drugs, linking ion channel inhibition to clinically relevant autonomic and cardiac effects [21,22,23]. In contrast, the Non-Selective Inhibition profile (Profile 2), defined by dose-independent blockade across tissues, suggests a non-competitive or high-affinity interaction, potentially consistent with calcium channel modulation, as described for several calcium channel modulators [24]. Such behavior is characteristic of several calcium channel modulators, which often interact with channel states or accessory sites and produce inhibition that is relatively insensitive to increasing agonist or depolarizing stimuli [24]. This interpretation is supported by the inclusion of the established calcium channel modulators flunarizine and cinnarizine within this group [24]. The Tissue-Dependent Inhibition profile (Profile 3) suggests more complex pharmacodynamic interactions, potentially reflecting tissue-specific target expression or differential drug accessibility, including variability in ion channel expression. In this context, tissue-specific target expression refers primarily to differences in the expression, density, or functional contribution of voltage-gated sodium and calcium channels, as well as associated regulatory proteins, across smooth muscle types [17]. Finally, the identification of a Minimal Inhibition profile (Profile 4) isolates compounds such as moclobemide that inherently lack significant membrane-stabilizing activity [25,26]. Collectively, this functional classification provides a coherent pharmacological framework to rationalize the differential direct effects of psychotherapeutic drugs on smooth muscle excitability. Importantly, direct confirmation of ion channel involvement was beyond the scope of the present study and warrants further investigation.

The functional profiles identified here demonstrate a notable concordance with the reported clinical burden of autonomic side effects associated with several psychotherapeutic drugs [21,27]. Agents within the High Inhibitory Liability profile, such as nortriptyline and paroxetine, are consistently reported to be associated with a higher incidence of autonomic adverse effects [27,28,29,30]. Nortriptyline, a prototypical tricyclic antidepressant, has been widely reported in association with orthostatic hypotension, constipation, and xerostomia—effects commonly attributed to its anticholinergic and membrane-stabilizing properties [7,22,23,30,31]. Similarly, paroxetine, despite belonging to the SSRI class, has been reported to exhibit a comparatively higher prevalence of anticholinergic-like side effects and poorer tolerability within its class [22,23,29,30,31]. This clinical observation is pharmacologically consistent with the direct inhibition of smooth muscle contraction observed in our ex vivo assay. In contrast, drugs classified in the Minimal Inhibition profile, most notably moclobemide, are clinically characterized by a low incidence of autonomic disturbances [25,26,32]. Taken together, these associations suggest the plausibility that direct suppression of smooth muscle excitability could represent a contributing pharmacodynamic factor to autonomic side-effect liability, complementing classical receptor-based explanations. However, this translational link remains hypothetical in the absence of direct correlation between the inhibitory potencies observed here and clinically achieved free drug concentrations.

The functional classification proposed here provides a pragmatic framework for contextualizing the direct smooth muscle effects of psychotherapeutic agents [32]. It may inform early-stage drug development by flagging compounds with a High Inhibitory Liability profile for more focused cardiovascular and autonomic safety evaluation [21,32]. The clinical relevance of this ex vivo profile is, however, context dependent. For instance, the direct inhibitory activity of reboxetine observed in our assay is likely counterbalanced in vivo by its potent noradrenergic effects, which may explain its association with hypertension rather than hypotension [27,33]. This highlights that the present model captures a fundamental pharmacodynamic vector whose net clinical impact is ultimately shaped by the drug’s integrated mechanism of action. Furthermore, the tissue-specificity observed for certain compounds underscores the value of multi-tissue profiling approaches. Future studies should aim to establish quantitative relationships between the inhibitory potencies reported here and (a) binding affinities for specific ion channels and (b) standardized adverse event rates derived from large pharmacovigilance databases, thereby advancing this functional taxonomy toward a more predictive translational tool. Notwithstanding these considerations, the functional taxonomy established here provides a novel, mechanism-informed lens through which to evaluate and contextualize the autonomic safety profile of psychotherapeutic drugs, offering a pragmatic tool for both basic pharmacology and early drug development.

Several limitations of the present study should be acknowledged. First, the study does not include direct experimental confirmation of the specific ion channels involved in the observed inhibitory effects, and the proposed mechanisms are therefore based on functional patterns and previously reported evidence. Second, not all compounds were uniformly evaluated across all smooth muscle preparations, reflecting a tiered experimental strategy influenced by ethical considerations and practical constraints. Third, the illustrative concentration–response curves were reconstructed from experimentally determined IC_50_ values and are provided for qualitative visualization rather than as original experimental recordings. Finally, the translational relevance of the findings remains to be established, as no direct correlation with clinically achieved drug concentrations was assessed. These limitations should be considered when interpreting the results and highlighting areas for future investigation.

## 4. Materials and Methods

### 4.1. Ethical Considerations

All experimental procedures involving animals were conducted in accordance with national and institutional guidelines for the care and use of laboratory animals applicable at the time of the study. All efforts were made to minimize animal suffering and to reduce the number of animals used. The animal study protocol was approved by the Institutional Research Ethics Committee of the University of Valladolid (protocol code PI 25-2409; 5 February 2025).

### 4.2. Animals and Tissue Preparation

Smooth muscle preparations were obtained from guinea-pig ileum, rat vas deferens, and rat uterus. Six male guinea pigs (300–500 g) and twelve Wistar rats (6 males and 6 females) (150–200 g) were used. Virgin female rats received a single subcutaneous injection of estradiol benzoate (0.5 mg/kg) 24 h prior to uterus extraction in order to standardize hormonal status [34].

Animals were anesthetized with isoflurane [35]. Target organs were rapidly excised and immediately placed in oxygenated physiological solution. Animals were subsequently euthanized by anesthetic overdose [35].

### 4.3. Isolated Organ Bath Setup and Solutions

Guinea-pig ileum was mounted in an organ bath containing Tyrode solution, continuously gassed with carbogen (95% O_2_/5% CO_2_), following established procedures [36,37].

Rat vas deferens was mounted in an organ bath containing Krebs–Henseleit solution, gassed with carbogen [36].

Rat uterus was mounted in an organ bath containing de Jalon solution [37].

All preparations were equilibrated under a resting tension appropriate for each tissue type until a stable baseline was achieved. Isometric contractions were recorded using standard force transduction systems [36,37].

### 4.4. Pharmacological Agents

All pharmacological agents were obtained from established chemical and pharmaceutical suppliers of analytical-grade compounds, including Sigma-Aldrich (Merck, St. Louis, MO, USA), MedChemExpress (MCE, Monmouth Junction, NJ, USA), Tocris Bioscience (Bristol, UK), LGC Standards (Teddington, UK), and BOC Sciences (Shirley, NY, USA). All compounds were of high purity (≥98%), as specified by the manufacturers and supported by certificates of analysis where available.

The following compounds were used: fluvoxamine maleate, fluoxetine hydrochloride, lofepramine hydrochloride, melitracen hydrochloride, adinazolam mesylate, moclobemide, sertraline hydrochloride, paroxetine hydrochloride, flunarizine hydrochloride, dotarizine, cinnarizine hydrochloride, clozapine, clotiapine, nortriptyline hydrochloride, nefazodone hydrochloride, citalopram hydrobromide, reboxetine mesylate, and risperidone.

Stock solutions were prepared in distilled water or dimethyl sulfoxide (DMSO), as appropriate. Final solvent concentrations in the organ bath did not exceed 0.1% (*v*/*v*), a concentration verified in control experiments to have no effect on basal tone or potassium ion (K^+^)-induced contraction.

### 4.5. Experimental Protocol for K^+^-Induced Contraction

In all preparations, a stable and reproducible smooth muscle contraction was elicited by increasing extracellular potassium concentration ([K^+^]o) to 105 mM using potassium chloride (KCl), corresponding to approximately a 20-fold increase above basal levels. This high-K^+^ stimulus reliably induces depolarization-dependent contraction independent of specific receptor activation [20,38].

Once a stable control contraction to 105 mM KCl was established, increasing concentrations of the test psychotherapeutic drug were added cumulatively to the organ bath. Responses were allowed to stabilize at each concentration (typically 5–10 min) before addition of the next concentration. Only one concentration–response curve was generated per tissue segment [36].

A tiered experimental strategy was employed: all drugs were initially evaluated in at least one smooth muscle preparation (typically rat uterus or vas deferens). Compounds exhibiting notable inhibitory activity were subsequently assessed in one or two additional preparations to evaluate dose-dependency and tissue consistency.

Drug concentration ranges were selected following a tiered pharmacological rationale. For each compound, concentrations were chosen to include (i) subthreshold levels with no detectable effect on K^+^-induced contraction, (ii) intermediate concentrations at which inhibition first became detectable (threshold concentrations), and (iii) higher concentrations sufficient to reveal maximal inhibition where present. This strategy allowed identification of inhibition thresholds, characterization of concentration–response relationships, and calculation of IC_50_ values when applicable, while also enabling discrimination between dose-dependent and dose-independent inhibitory patterns. Because the experiments were performed under controlled ex vivo conditions, systemic pharmacokinetic parameters were not considered, as they are not applicable to this experimental context. This experimental design ensured that all compounds were evaluated across multiple concentrations, enabling systematic comparison of inhibitory behavior across drug classes and smooth muscle preparations.

### 4.6. Illustrative Concentration–Response Curves

For selected compounds, illustrative concentration–response curves were generated using a standard sigmoidal model parameterized with experimentally determined IC_50_ values obtained in the present study. These curves were constructed exclusively to facilitate visualization of relative inhibitory potency and profile shape across compounds and tissues. They do not represent original experimental concentration–response recordings, do not include experimental variability, and were not used for quantitative analysis or IC_50_ determination.

### 4.7. Data and Statistical Analysis

Contractile responses were expressed as a percentage of the control contraction induced by 105 mM potassium chloride. For drugs producing concentration-dependent inhibition, half-maximal inhibitory concentrations (IC_50_) and their 95% confidence intervals were calculated according to the principles of Litchfield and Wilcoxon [39], using appropriate concentration–response curve fitting methods.

Drugs exhibiting dose-independent inhibition (i.e., absence of a sigmoidal concentration–response relationship) or no detectable effect within the concentration range tested were described qualitatively.

The sample size (*n*) for each concentration–response curve represents the number of independent animals, with one tissue preparation obtained per animal. For the majority of experiments, *n* = 6. Data are presented as IC_50_ values with 95% confidence intervals or as qualitative descriptors, as detailed in Table 1.

## 5. Conclusions

This study presents a systematic comparative pharmacological profiling of a broad panel of psychotherapeutic drugs based on their direct effects on smooth muscle excitability, independent of classical receptor-mediated mechanisms. Using a standardized KCl-induced depolarization assay, we reveal pronounced heterogeneity in inhibitory activity, enabling the stratification of these agents into four distinct functional profiles: High Inhibitory Liability, Non-Selective Inhibition, Tissue-Dependent Inhibition, and Minimal Inhibition.

This functional taxonomy captures a fundamental pharmacodynamic dimension of psychotherapeutic drugs beyond classical receptor blockade. Notably, the High Inhibitory Liability profile corresponds to agents clinically associated with a substantial burden of autonomic adverse effects, including orthostatic hypotension and constipation. This concordance supports the plausibility that direct suppression of smooth muscle excitability contributes to autonomic side-effect liability, complementing established receptor-based mechanisms.

Accordingly, the classification framework proposed here provides a pragmatic, mechanism-informed perspective for contextualizing the autonomic safety profile of psychotherapeutic drugs. It may support early-stage drug development by identifying compounds with a high intrinsic potential for direct smooth muscle interaction, thereby guiding more focused safety evaluations and facilitating the rational selection of agents with improved autonomic tolerability.

## Figures and Tables

**Table 1:** Comparative inhibitory potency of psychotherapeutic drugs on KCl-induced smooth muscle contraction (ex vivo model).

Drug	Rat Uterus IC_50_ (M) [95% CI]	Rat Vas Deferens IC_50_ (M) [95% CI]	Guinea-Pig Ileum IC_50_ (M) [95% CI]	Dose-Dependency
Fluvoxamine	1.82 × 10^−5^ (1.24–4.26 × 10^−5^)	NT	NT	Dose-dependent
Fluoxetine	Dose-independent inhibition	NT	NT	Dose-independent
Clotiapine	3.97 × 10^−6^ (2.68–4.69 × 10^−6^)	1.95 × 10^−6^ (1.09–2.19 × 10^−6^)	NT	Dose-dependent
Lofepramine	3.40 × 10^−6^ (2.53–4.59 × 10^−6^)	3.75 × 10^−5^ (2.35–4.95 × 10^−5^)	NT	Dose-dependent
Clozapine	1.27 × 10^−5^ (1.08–2.22 × 10^−5^)	7.76 × 10^−5^ (4.59–8.90 × 10^−5^)	NT	Dose-dependent
Melitracen	1.20 × 10^−5^ (0.92–2.39 × 10^−5^)	2.47 × 10^−6^ (1.36–2.89 × 10^−6^)	NT	Dose-dependent
Moclobemide	No effect	No effect	NT	No inhibition
Sertraline	6.77 × 10^−5^ (5.22–8.70 × 10^−5^)	8.97 × 10^−5^ (6.38–9.96 × 10^−5^)	NT	Dose-dependent
Adinazolam	1.48 × 10^−4^ (1.08–2.74 × 10^−4^)	3.51 × 10^−5^ (2.59–4.86 × 10^−5^)	NT	Dose-dependent
Paroxetine	7.17 × 10^−6^ (4.24–8.25 × 10^−6^)	9.45 × 10^−6^ (8.34–10.7 × 10^−6^)	NT	Dose-dependent
Cinnarizine	5.85 × 10^−6^ (4.59–7.20 × 10^−6^)	Dose-independent inhibition	Dose-independent inhibition	Dose-independent
Dotarizine	Dose-independent inhibition	Dose-independent inhibition	Dose-independent inhibition	Dose-independent
Flunarizine	Dose-independent inhibition	Dose-independent inhibition	Dose-independent inhibition	Dose-independent
Nortriptyline	2.50 × 10^−6^ (1.90–3.20 × 10^−6^)	6.90 × 10^−7^ (6.20–7.30 × 10^−7^)	1.70 × 10^−5^ (1.60–1.80 × 10^−5^)	Dose-dependent
Citalopram	6.08 × 10^−5^ (6.00–8.10 × 10^−5^)	6.72 × 10^−6^ (2.47–7.00 × 10^−6^)	4.85 × 10^−5^ (3.80–5.00 × 10^−5^)	Dose-dependent
Reboxetine	7.60 × 10^−6^ (6.90–8.00 × 10^−6^)	No effect	No effect	Tissue-dependent
Risperidone	No effect	7.50 × 10^−6^ (6.38–8.96 × 10^−6^)	Dose-independent inhibition	Tissue-dependent
Nefazodone	6.23 × 10^−6^ (5.62–7.00 × 10^−6^)	4.30 × 10^−5^ (3.74–4.87 × 10^−5^)	5.56 × 10^−6^ (5.10–6.00 × 10^−6^)	Dose-dependent

Dose-independent inhibition: Inhibition observed but not following a classical sigmoidal concentration–response relationship, precluding IC_50_ calculation. No effect: Tested at relevant concentrations with no detectable modification of K^+^-induced contraction. IC_50_ values are presented with their 95% confidence intervals where calculable. The selection of tissues for each compound was based on experimental feasibility, including tissue availability, preparation stability over prolonged recording periods, and the need to minimize animal use, rather than on differences in intrinsic sensitivity between preparations. When a tissue is marked as NT, the corresponding compound was not evaluated in that preparation. To further support visual comparison of inhibitory behavior across tissues, illustrative concentration–response curves reconstructed from experimentally determined IC_50_ values are provided as Supplementary Figures S1–S3.

**Table 2:** Functional pharmacological profiling of psychotherapeutic drugs based on inhibitory patterns.

Profile Category	Criteria	Representative Drugs	Pharmacodynamic Implication
High Inhibitory Liability	Low micromolar IC_50_, dose-dependent inhibition in ≥2 tested tissues	Nortriptyline, Clotiapine, Paroxetine, Nefazodone, Melitracen	High, consistent interference with membrane excitability, potentially involving inhibition of voltage-gated sodium (Na^+^) and/or calcium (Ca^2+^) channels
Non-Selective Inhibition	Dose-independent inhibition across all tested tissues	Flunarizine, Dotarizine, Cinnarizine	Likely Ca^2+^ channel-related effects or non-competitive, high-affinity interactions
Tissue-Dependent Inhibition	Inhibition restricted to specific tissues	Risperidone, Reboxetine	Context-dependent pharmacodynamics, potentially reflecting tissue-specific expression of ion channels or differential drug accessibility
Minimal or Absent Inhibition	No detectable effect in tested preparations	Moclobemide	Low inherent interaction with voltage-gated ion channels

Mechanistic implications are proposed based on functional inhibition patterns and previously reported ion channel interactions, and do not imply direct molecular confirmation.

**Table 3:** Summary of tissue consistency of inhibitory effects for selected compounds.

Drug	Rat Uterus	Rat Vas Deferens	Guinea-Pig Ileum	Tissue Consistency
Nortriptyline	✓ (IC_50_)	✓ (IC_50_)	✓ (IC_50_)	High
Cinnarizine	✓ (IC_50_)	✓ (Dose-indep.)	✓ (Dose-indep.)	High
Flunarizine	✓ (Dose-indep.)	✓ (Dose-indep.)	✓ (Dose-indep.)	High
Risperidone	✗ (No effect)	✓ (IC_50_)	✓ (Dose-indep.)	Low (Tissue-dependent)
Reboxetine	✓ (IC_50_)	✗ (No effect)	✗ (No effect)	Low (Tissue-dependent)
Moclobemide	✗ (No effect)	✗ (No effect)	NT	None

✓: Inhibition observed; ✗: No inhibition observed; NT: Not tested. The nature of inhibition (IC_50_ or dose-independent) is specified in parentheses.

## Data Availability

The original contributions presented in this study are included in the article/Supplementary Material. Further inquiries can be directed to the corresponding authors.

## Supplementary Materials

The following supporting information can be downloaded at: https://www.mdpi.com/article/10.3390/ph19040645/s1. Supplementary Figure S1: Illustrative concentration–response curves reconstructed from experimentally determined IC_50_ values for psychotherapeutic drugs in rat uterus. Curves were generated using a standard sigmoidal model with identical slope parameters and are intended solely as conceptual visualizations to facilitate comparison of relative inhibitory potency. These curves do not represent original experimental recordings. Supplementary Figure S2. Illustrative concentration–response curves reconstructed from experimentally determined IC_50_ values for psychotherapeutic drugs in rat vas deferens. Curves were generated using a standard sigmoidal model with identical slope parameters and are intended solely as conceptual visualizations to facilitate comparison of relative inhibitory potency. These curves do not represent original experimental recordings. Supplementary Figure S3. Illustrative concentration–response curves reconstructed from experimentally determined IC_50_ values for psychotherapeutic drugs in guinea-pig ileum. Curves were generated using a standard sigmoidal model with identical slope parameters and are intended solely as conceptual visualizations to facilitate comparison of relative inhibitory potency. These curves do not represent original experimental recordings.

## References

[1] Hilmer S.N., Gnjidic D. (2022). The Anticholinergic Burden: From Research to Practice. Aust. Prescr..

[2] Stroup T.S., Gray N. (2018). Management of Common Adverse Effects of Antipsychotic Medications. World Psychiatry.

[3] Milan R., Vasiliadis H.-M. (2020). The Association between Side Effects and Adherence to Antidepressants among Primary Care Community-Dwelling Older Adults. Aging Ment. Health.

[4] Kikuchi T., Suzuki T., Uchida H., Watanabe K., Mimura M. (2013). Association between Antidepressant Side Effects and Functional Impairment in Patients with Major Depressive Disorders. Psychiatry Res..

[5] Korchia T., Faugère M., Achour V., Maakaron E., Andrieu-Haller C., Fond G., Lançon C. (2025). Impact of Perceived Side-Effects of Psychotherapeutic Treatments on Quality of Life in Patients with Severe Mental Illness. Dialogues Clin. Neurosci..

[6] Alvares G.A., Quintana D.S., Hickie I.B., Guastella A.J. (2016). Autonomic Nervous System Dysfunction in Psychiatric Disorders and the Impact of Psychotherapeutic Medications: A Systematic Review and Meta-Analysis. J. Psychiatry Neurosci..

[7] Rivasi G., Rafanelli M., Mossello E., Brignole M., Ungar A. (2020). Drug-Related Orthostatic Hypotension: Beyond Anti-Hypertensive Medications. Drugs Aging.

[8] Trinchieri M., Perletti G., Magri V., Stamatiou K., Montanari E., Trinchieri A. (2021). Urinary Side Effects of Psychotherapeutic Drugs: A Systematic Review and Metanalysis. Neurourol. Urodyn..

[9] Bhanu C., Nimmons D., Petersen I., Orlu M., Davis D., Hussain H., Magammanage S., Walters K. (2021). Drug-Induced Orthostatic Hypotension: A Systematic Review and Meta-Analysis of Randomised Controlled Trials. PLoS Med..

[10] Seeman P. (1966). Membrane Stabilization by Drugs: Tranquilizers, Steroids, and Anesthetics. Int. Rev. Neurobiol..

[11] Nau C., Seaver M., Wang S.-Y., Wang G.K. (2000). Block of Human Heart HH1 Sodium Channels by Amitriptyline. J. Pharmacol. Exp. Ther..

[12] Wang G.K., Russell C., Wang S.-Y. (2004). State-Dependent Block of Voltage-Gated Na^+^ Channels by Amitriptyline via the Local Anesthetic Receptor and Its Implication for Neuropathic Pain. Pain.

[13] Lenkey N., Károly R., Kiss J.P., Szász B.K., Vízi E.S., Mike A. (2006). The Mechanism of Activity-Dependent Sodium Channel Inhibition by the Antidepressants Fluoxetine and Desipramine. Mol. Pharmacol..

[14] Imbrici P., Conte Camerino D., Tricarico D. (2013). Major Channels Involved in Neuropsychiatric Disorders and Therapeutic Perspectives. Front. Genet..

[15] Zamponi G.W. (2016). Targeting Voltage-Gated Calcium Channels in Neurological and Psychiatric Diseases. Nat. Rev. Drug Discov..

[16] Ogata N., Narahashi T. (1989). Block of Sodium Channels by Psychotherapeutic Drugs in Single Guinea-Pig Cardiac Myocytes. Br. J. Pharmacol..

[17] Hill-Eubanks D.C., Werner M.E., Heppner T.J., Nelson M.T. (2011). Calcium Signaling in Smooth Muscle. Cold Spring Harb. Perspect. Biol..

[18] Sanders K.M. (2008). Regulation of Smooth Muscle Excitation and Contraction. Neurogastroenterol. Motil..

[19] Becker B., Morel N., Vanbellinghen A.-M., Lebrun P. (2004). Blockade of Calcium Entry in Smooth Muscle Cells by the Antidepressant Imipramine. Biochem. Pharmacol..

[20] Karaki H., Urakawa N., Kutsky P. (1984). Potassium-Induced Contraction in Smooth Muscle. Nihon Heikatsukin Gakkai Zasshi.

[21] Mackin P. (2008). Cardiac Side Effects of Psychiatric Drugs. Hum. Psychopharmacol. Clin. Exp..

[22] Cusack B., Nelson A., Richelson E. (1994). Binding of Antidepressants to Human Brain Receptors: Focus on Newer Generation Compounds. Psychopharmacology.

[23] Richelson E. (1994). Pharmacology of Antidepressants—Characteristics of the Ideal Drug. Mayo Clin. Proc..

[24] Holmes B. (1984). Flunarizine. A Review of Its Pharmacodynamic and Pharmacokinetic Properties and Therapeutic Use. Drugs.

[25] Siepmann M. (2004). The Effects of Moclobemide on Autonomic and Cognitive Functions in Healthy Volunteers. Pharmacopsychiatry.

[26] Versiani M. (1990). Tolerability of Moclobemide, a New Reversible Inhibitor of Monoamine Oxidase-A, Compared with Other Antidepressants and Placebo. Acta Psychiatr. Scand..

[27] Calvi A. (2021). Antidepressant Drugs Effects on Blood Pressure. Front. Cardiovasc. Med..

[28] Derry S., Wiffen P.J., Aldington D., Moore R.A. (2015). Nortriptyline for Neuropathic Pain in Adults. Cochrane Database Syst. Rev..

[29] Westenberg H.G.M., Sandner C. (2006). Tolerability and Safety of Fluvoxamine and Other Antidepressants. Int. J. Clin. Pract..

[30] Fujishiro J., Imanishi T., Onozawa K., Tsushima M. (2002). Comparison of the Anticholinergic Effects of the Serotonergic Antidepressants, Paroxetine, Fluvoxamine and Clomipramine. Eur. J. Pharmacol..

[31] Cipriani A., Furukawa T.A., Salanti G., Chaimani A., Atkinson L.Z., Ogawa Y., Leucht S., Ruhe H.G., Turner E.H., Higgins J.P.T. (2018). Comparative Efficacy and Acceptability of 21 Antidepressant Drugs for the Acute Treatment of Adults with Major Depressive Disorder: A Systematic Review and Network Meta-Analysis. Lancet.

[32] Chaudhary K.W., Clancy C.E., Yang P.-C., Pierson J.B., Goldin A.L., Koerner J.E., Wisialowski T.A., Valentin J.-P., Imredy J.P., Lagrutta A. (2024). An Overview of Drug-Induced Sodium Channel Blockade and Changes in Cardiac Conduction: Implications for Drug Safety. Clin. Transl. Sci..

[33] Penttilä J. (2001). The Effects of Amitriptyline, Citalopram and Reboxetine on Autonomic Nervous System: A Randomised Placebo-Controlled Study on Healthy Volunteers. Psychopharmacology.

[34] Watcho P., Ngadjui E., Alango Nkeng-Efouet P., Nguelefack T.B., Kamanyi A. (2011). Evaluation of in Vitro Uterotonic Activities of Fruit Extracts of Ficus Asperifolia in Rats. Evid.-Based Complement. Altern. Med..

[35] Oh S.S., Narver H.L. (2024). Mouse and Rat Anesthesia and Analgesia. Curr. Protoc..

[36] Upchurch W.J., Iaizzo P.A. (2022). In Vitro Contractile Studies within Isolated Tissue Baths: Translational Research from Visible Heart^®^ Laboratories. Exp. Biol. Med..

[37] Premrov Bajuk B., Prem L., Vake T., Žnidaršič N., Snoj T. (2022). The Effect of Thymol on Acetylcholine-Induced Contractions of the Rat Ileum and Uterus under Ex Vivo Conditions. Front. Pharmacol..

[38] Ratz P.H., Berg K.M., Urban N.H., Miner A.S. (2005). Regulation of Smooth Muscle Calcium Sensitivity: KCl as a Calcium-Sensitizing Stimulus. Am. J. Physiol. Physiol..

[39] Litchfield J.T., Wilcoxon F. (1949). A Simplified Method of Evaluating Dose-Effect Experiments. J. Pharmacol. Exp. Ther..

