# Supplementary material for: Comparative Pharmacological Profiling of Psychotherapeutic Drugs Reveals a Functional Taxonomy Based on Direct Inhibition of Smooth Muscle Excitability
Source: Pharmaceuticals (Basel). 2026 Apr 21;19(4):645. doi: 10.3390/ph19040645 (PMC13118299; doi:10.3390/ph19040645)
Supplement: Supplementary file 1 [file pharmaceuticals-19-00645-s001.zip › pharmaceuticals-4245916-supplementary.pdf]

Supplementary Figures

Supplementary Figure S1. Illustrative concentration–response curves reconstructed from IC_{50} values in rat uterus

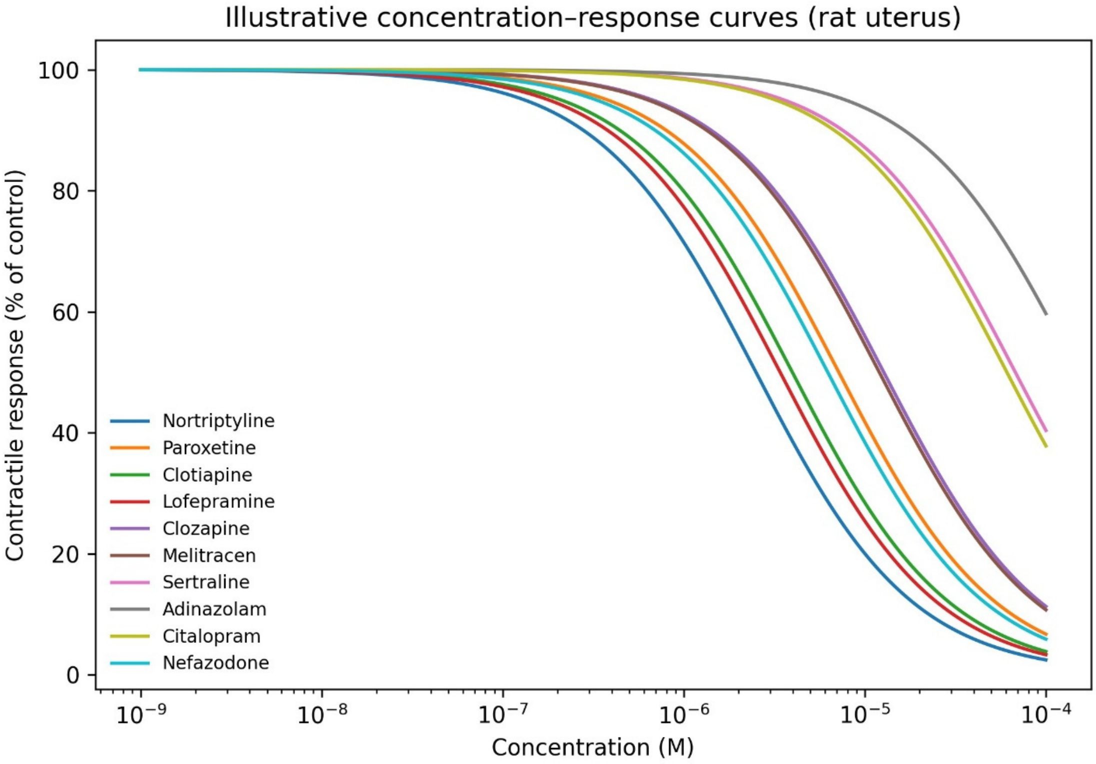

Supplementary Figure S1: Illustrative concentration–response curves reconstructed from experimentally determined IC_{50} values for psychotherapeutic drugs in rat uterus. Curves were generated using a standard sigmoidal model with identical slope parameters and are intended solely as conceptual visualizations to facilitate comparison of relative inhibitory potency. These curves do not represent original experimental recordings.

Supplementary Figure S2. Illustrative concentration–response curves reconstructed from IC_{50} values in rat vas deferens

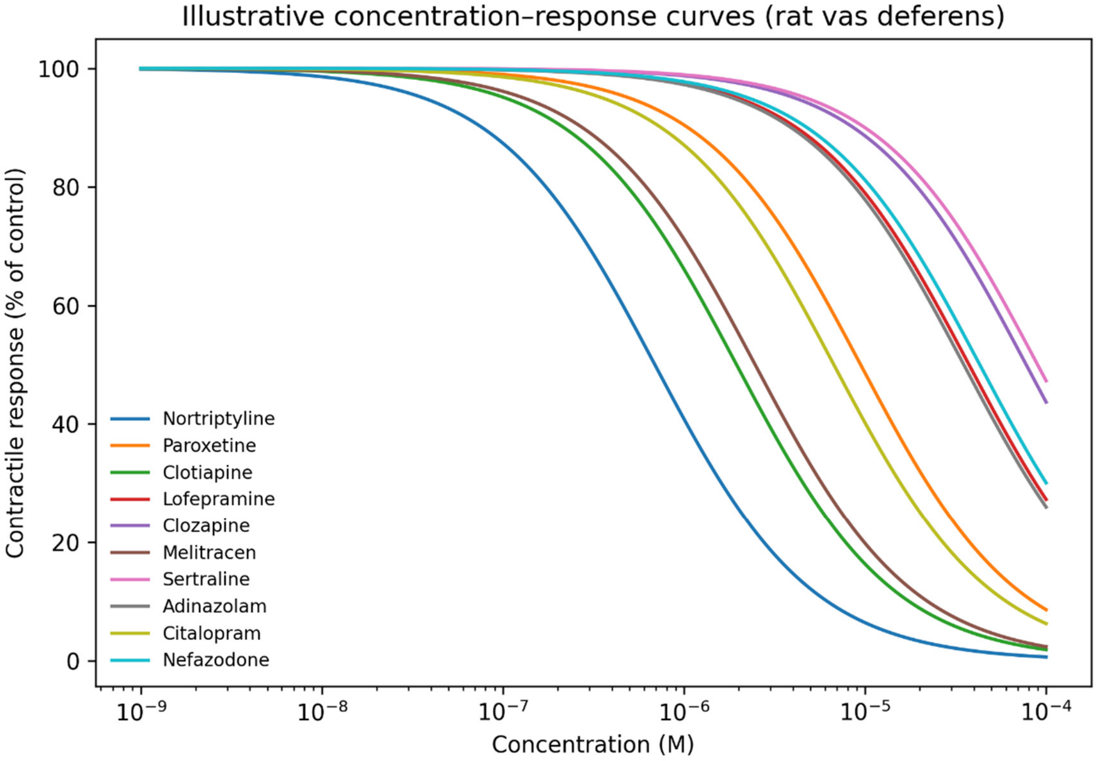

Supplementary Figure S2. Illustrative concentration–response curves reconstructed from experimentally determined IC_{50} values for psychotherapeutic drugs in rat vas deferens. Curves were generated using a standard sigmoidal model with identical slope parameters and are intended solely as conceptual visualizations to facilitate comparison of relative inhibitory potency. These curves do not represent original experimental recordings.

Supplementary Figure S3. Illustrative concentration–response curves reconstructed from IC_{50} values in guinea-pig ileum

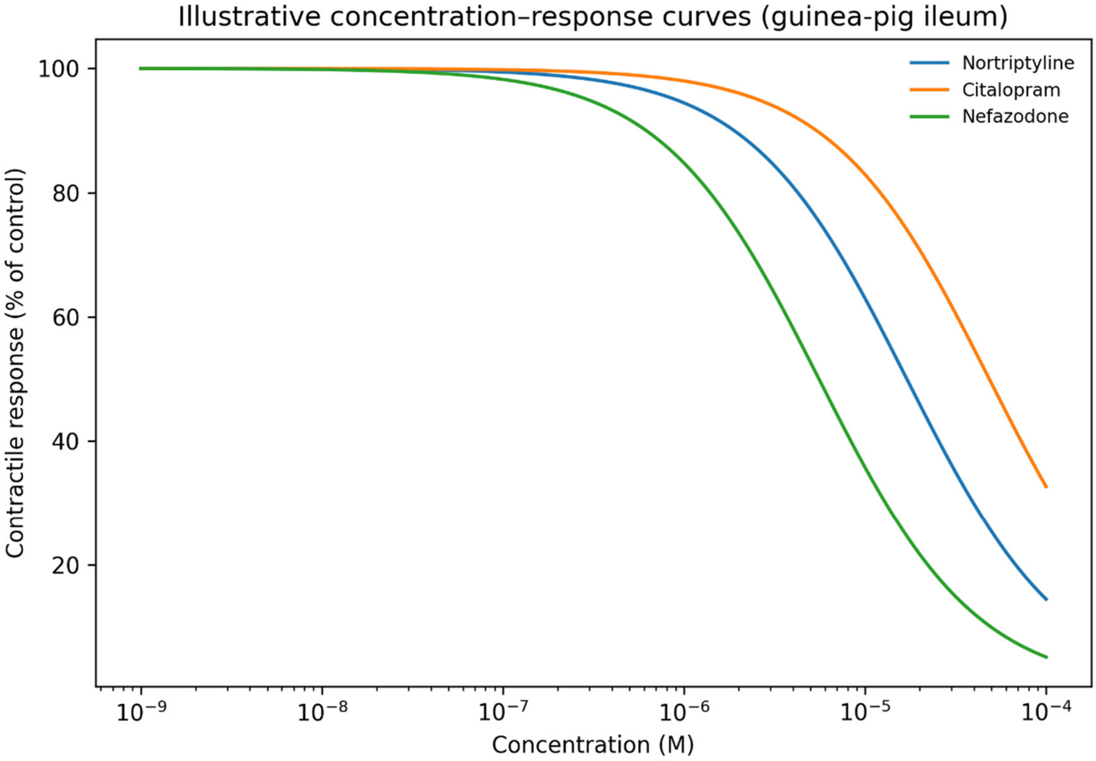

Supplementary Figure S3. Illustrative concentration–response curves reconstructed from experimentally determined IC_{50} values for psychotherapeutic drugs in guinea-pig ileum. Curves were generated using a standard sigmoidal model with identical slope parameters and are intended solely as conceptual visualizations to facilitate comparison of relative inhibitory potency. These curves do not represent original experimental recordings.
